# One-year follow-up comparison of two hybrid closed-loop systems in Italian children and adults with type 1 diabetes

**DOI:** 10.3389/fendo.2023.1099024

**Published:** 2023-01-26

**Authors:** Marta Bassi, Luca Patti, Irene Silvestrini, Marina Francesca Strati, Marta Ponzano, Nicola Minuto, Davide Maggi

**Affiliations:** ^1^ Department of Neuroscience, Rehabilitation, Ophthalmology, Genetics, Maternal and Child Health, University of Genoa, Genoa, Italy; ^2^ Department of Pediatrics, IRCCS Istituto Giannina Gaslini, University of Genoa, Genoa, Italy; ^3^ Department of Internal Medicine and Medical Specialties (DiMI), University of Genoa, Genoa, Italy; ^4^ Department of Health Science (DiSSAL), University of Genoa, Genoa, Italy; ^5^ Diabetes Clinic, IRCCS Ospedale Policlinico San Martino Genoa, Genoa, Italy

**Keywords:** AHCL (Advanced Hybrid Closed Loop), type 1 diabetes, CGM – continous glucose monitoring, TIR (time in range), CSII - continuous subcutaneous insulin infusion

## Abstract

**Background and aims:**

Tandem Control-IQ and MiniMed 780G are the main Advanced Hybrid Closed Loop (AHCL) systems currently available in pediatric and adult patients with Type 1 Diabetes (T1D). The aim of our study was to evaluate glycemic control after 1-year of follow-up extending our previous study of 1-month comparison between the two systems.

**Methods:**

We retrospectively compared clinical and continuous glucose monitoring (CGM) data from the patients included in the previous study which have completed 1-year observation period. The study population consisted of 74 patients, 42 Minimed 780G users and 32 Tandem Control-IQ users. Linear mixed models with random intercept were performed to study the variations over time and the interaction between time and system; Mann-Whitney or T-test were used to compare systems at 1-year.

**Results:**

Both systems have been shown to be effective in maintaining the glycemic improvement achieved one month after starting AHCL. Significant changes over time were observed for TIR, TAR, TAR>250mg/dl, average glucose levels and SD (p<0.001). At 1-year follow-up Minimed 780G obtained better improvement in TIR (p<0.001), TAR (p=0.002), TAR>250mg/dl (p=0.001), average glucose levels (p<0.001). The comparison of the glycemic parameters at 1-year showed a significant superiority of Minimed 780G in terms of TIR (71% vs 68%; p=0.001), TAR (p=0.001), TAR>250 (p=0.009), average glucose levels(p=0.001) and SD (p=0.031).

**Conclusions:**

The use of AHCL systems led to a significant improvement of glycemic control at 1-month, which is maintained at 1-year follow-up. MiniMed is more effective than Tandem in reaching the International recommended glycemic targets. Continuous training and education in the use of technology is essential to get the best out of the most advanced technological tools.

## 1 Introduction

The management of type 1 diabetes (T1D) has changed substantially over the past ten years. Evolving technologies offer the potential to highly improve glycemic control. Systems which integrate insulin infusion with continuous glucose monitoring (CGM) are now widely used by T1D patients (1–5).

Advanced Hybrid Closed Loop (AHCL) systems combine automated basal rate and correction boluses to keep glycemic values in a target range. Patients are only required to estimate carbohydrate consumption for meal boluses (6, 7). In Italy two AHCL systems are provided for both adult and pediatric populations by the national health system: the Tandem t:slim X2 Control IQ™ system (Tandem Inc., San Diego, California); and the Minimed™ 780G system (Minimed Medtronic, Northridge, California). The Minimed 780G pump is integrated with the Guardian Sensor 4 (Medtronic, Northridge, California), the Tandem Control-IQ is associated with the Dexcom G6 (Dexcom Inc., San Diego, CA) system.

These two systems use different algorithms for basal rate infusion and correction boluses and different glycemic targets. Minimed 780g uses a PID (proportional-integrative-derivative) algorithm. This algorithm adjusts the insulin infusion based on the glycemic trend of the previous few minutes, evaluating: the difference between blood glucose levels measured at in a certain moment and the blood glucose target (proportional component), the difference between the area under the curve of the measured blood glucose level and the blood glucose “target” (integral component) and the speed and direction of change in glucose values ​​(derivative component). Tandem Control-IQ uses a model predictive control (MPC) algorithm. This algorithm predicts glucose levels in the future by minimizing the difference between predicted glucose values and those measured in a given period of time, it “learns” how to autonomously respond to glycemic changes with optimal insulin infusion regimens and it is proactive (anticipates the glucose-lowering effect of insulin).

Minimed 780G can carry out up to 12 correction boluses per hour and decide the basal rate automatically. Control-IQ system is able to deliver a maximum of one correction bolus per hour and modifies the basal profile based on a 30-minute prediction horizon of glucose levels. Both systems have special modes dedicated to sport and physical activity and Control-IQ has a Sleep mode with a narrower target range. Furthermore, Minimed 780G system automatically calculates the total daily insulin need in order to define the insulin sensitivity factor (ISF); the patient can only customize the insulin-to-carbohydrate (I/CHO) ratios for meal boluses, the active insulin time (AIT) and the glycemic target used by the algorithm (SmartGuard). Control-IQ system uses fixed AIT of 5h; the user can change the basal rate, ISF and I/CHO ratios for meal boluses.

Currently, the CGM parameters indicating a good glycemic control are defined by the International Consensus as: Time in Range (TIR) (70-180 mg/dl) > 70%, Time Below Range (TBR) (<70 mg/dl) < 4%, TBR<54 mg/dl < 1%, Time Above Range (TAR) (>180 mg/dl) < 25%, TAR>250 mg/dl <1% (8, 9).

Early studies on the use of Tandem Control-IQ or Minimed 780G in adolescents and adults with type 1 diabetes have shown excellent results in terms of glycemic outcomes and patient satisfaction (10, 11). The results of 6-month and 1-year real-world use of Tandem Control-IQ system confirmed the conclusions reached by the pivotal trial, showing an increase in time in range (TIR 70–180 mg/dl) up to 73.5% at 12 months in a large sample of T1D patients (12, 13). Several multicenter studies conducted in children, adolescents and adults demonstrated the efficacy of Control-IQ compared to sensor-augmented pumps (14–16) and to PLGS algorithm (17). Two recent studies have demonstrated the efficacy of Tandem Control-IQ even in T1D patients with poor baseline glycemic control and in T2D (Type 2 Diabetes) and regardless of users’ engagement with the system or type of medical insurance (18–20).

Likewise, the use of Minimed 780G system has shown to be safe and effective and leads to an improvement of glycemic control in both the adult and pediatric populations and regardless of previous insulin strategy and baseline glucose control (21–28). A recent real-world study on 6-month-use of Minimed in more than 12000 adult and pediatric T1D patients showed that more than 75% of users achieved international consensus-recommended glycemic control (29).

Despite the evidence on the efficacy of ACHL systems, there are only two clinical studies comparing data on benefits and glycemic outcomes after 1-month of use of Minimed 780G and Tandem Control-IQ. In both studies, the use of AHCL systems led to a significant improvement of glycemic control (30, 31). The first study involved 90 adult and pediatric patients and results showed Minimed more effective in managing hyperglycemia and Tandem more effective in reducing hypoglycemia (30). Schiaffini et al. compared the two AHCL systems in 31 pediatric patients and their results did not show significant differences in glycemic control between the two systems (31). To our knowledge, there are no clinical studies comparing data on benefits and glycemic outcomes after a longer follow-up.

The aim of our study was to evaluate glycemic control after 1-year of follow-up extending our previous study of 1-month comparison between the two systems (30).

## 2 Materials and methods

A retrospective dual center study was performed from October 2020 to October 2021. A total of 90 T1D patients, followed at the IRCCS G.Gaslini Pediatric Diabetology Center (Genoa, Italy) or San Martino Polyclinic Hospital Diabetes Clinic (Genoa, Italy), were upgraded to Minimed 780G or Tandem Control-IQ. This is a follow-up study; results from the previous one-month comparison study have already been published (30).

Patients were enrolled according to the following inclusion criteria: T1D diagnosis at least one-year prior to the study, insulin therapy with CSII or MDI, use of CGM with at least one-months’ worth of data before and after starting the AHCL. Patients who dropped out of the AHCL system before one year of use and/or of whom we were unable to download glycemic data at T2 were excluded. Patients who were affected by other types of diabetes or had been using AHCL systems since disease onset were also excluded.

The observational period was divided in Time 0 (T0 – first use AHCL) and Time 2 (T2 – one year of ACHL therapy). At T0, the following data were collected for each patient: demographical data (sex, date of birth, age), age at clinical onset of T1D, duration of disease, previous type of insulin therapy, glycated hemoglobin value and general glycemic control data. At T0 and T2 we compared: glycated hemoglobin (HbA1c) values, and blood glucose control data of the previous 14 days, through the CGM data download. The following parameters were evaluated: TIR, TAR, TAR > 250 mg/dl, TBR, TBR < 54 mg/dl, Coefficient of Variation (CV), Standard Deviation (SD) and time of sensor use. The analysis at T2 was performed with both systems in Automatic Mode (Control-IQ or SmartGuard). CGM data were collected using data download platforms based on the technology used.

All patients (or parents if age < 18 years) provided a written informed consent in accordance with EU regulation 2016/679 to participate in the study.

### 2.1 Statistical analysis

Results were reported as median with interquartile range (IQR) for continuous variables and as absolute frequency with percentage for categorical variables, overall and by treatment group.

Comparisons of the baseline characteristics between the two treatment groups were assessed performing Chi-squared or Fisher’s exact test (categorical variables) and T-test or Mann-Whitney test (continuous variables) depending on the distribution of the variables.

All the parameters at T0 and T2 were studied performing linear mixed models with random intercept and adjusted for the following baseline variables: age, disease duration, HbA1c and type of previous treatment. To compare the pattern change between the two systems, the interaction between time and system was tested. Transformations were made for some variables due to a skewed distribution (graphically evaluated using histograms and graphs of quantiles against the quantiles of normal distribution). As a sensitivity analysis, the time*system interaction was investigated separately within the subsample of pediatric (age<18 years) and of adult (>=18 years) patients. Additionally, at T2, all the parameters were compared between the two groups using T-test or Mann-Whitney test depending on the distribution of the variables.

Missing data were not imputed, and a complete-case analysis was performed. A two- sided α less than 0.05 was considered statistically significant. All statistical analysis was performed using Stata version 16.0 (Stata Corporation, College Station, TX, USA).

## 3 Results

We collected the data of 74 patients (38 males, 36 females) from two Regional Pediatric (63 patients) and Adult (11 patients) Diabetology Centers (IRCCS G.Gaslini and San Martino Polyclinic Hospital, Genoa, Liguria). 42 of these patients used the Minimed 780G system and 32 the Tandem-Control IQ system. 16 patients, part of the initial trial, were excluded from this extended one because data download was unavailable at T2. The clinical characteristics of the population at baseline (T0) are summarized in **Table 1**, overall and divided by type of treatment.

**Table 1:** Patient characteristics at baseline (T0), overall and by treatment group.

	Overall N = 74 (100%)	Minimed 780G N = 42 (57%)	Control-IQ N = 32 (43%)	p-value
Male, N (%)	38 (51%)	22 (52%)	16 (50%)	0.8390
Age, Median (IQR)	17.2 (11.5; 26.1)	22.1 (11.8; 31.0)	15.5 (10.5; 19.9)	0.0141
Disease duration (yrs), Median (IQR)	9.8 (4.5; 17.4)	13.0 (5.0; 19.7)	7.4 (2.4; 11.0)	0.0133
HbA1c (%), Median (IQR)	7.4 (7; 7.8)	7.6 (7.2; 8)	7.3 (6.7; 7.7)	0.0015
TIR (%), Median (IQR)	55 (45; 63)	53.5 (43; 63)	55.5 (49.5; 66)	0.0905
TAR (%), Median (IQR)	27 (21; 34)	28.5 (22; 34)	26 (21; 30)	0.0855
TAR250 (%), Median (IQR)	13 (6; 23)	13.5 (6; 25)	12.5 (6.5; 18)	0.5468
TBR (%), Median (IQR)	1 (1; 4)	1.5 (1; 4)	1 (1; 4)	0.5184
TBR54 (%), Median (IQR)	0.1 (0; 1)	0.1 (0; 1)	0.2 (0; 1)	0.7073
Average glucose (mg/dl), Median (IQR)	174 (161; 190)	177 (162; 195)	174 (151.5; 181.5)	0.1970
SD (mg/dl), Median (IQR)	62.5 (51; 71)	60.5 (51; 74.5)	64 (53; 67)	0.6278
CV (%), Median (IQR)	36 (33; 40)	35 (31.3; 39)	37.6 (34.6; 40.7)	0.2584
Time Active CGM (%), Median (IQR)	95 (88; 98.3)	92.9 (85; 96)	98 (95; 98.9)	0.0005
Previous treatment, N (%)				<0.001
MDI	13 (18%)	7 (17%)	6 (19%)	
SAP	18 (24%)	11 (26%)	7 (22%)	
PLGS	28 (38%)	9 (21%)	19 (59%)	
HCL	15 (20%)	15 (36%)	0 (0%)	

IQR, Interquartile Range; HbA1c - Glycated Hemoglobin; TIR, Time in Range (70-180 mg/dl); TAR, Time Above Range (181-250 mg/dl); TAR250, Time Above Range (>250 mg/dl); TBR, Time Below Range (54-69 mg/dl); TBR54, Time Below Range (<54 mg/dl); SD, Standard Deviation; CV, Coefficient of Variation; CGM, Continuous Glucose Monitoring; MDI, Multiple Daily Injections; SAP, Sensor Augmented Pump; PLGS, Predictive Low Glucose Suspend; HCL, Hybrid Closed Loop. Bold values indicates statistically significant.

The median age of our population was 17.2 years (IQR=11.5; 26.1): Control-IQ users were younger (median age 15.5 years vs 22.1; p=0.0141) and had shorter disease duration (median 7.4 years vs 13.0; p = 0.0133). Patients in Control-IQ group compared to patients in Minimed 780G group had lower baseline HbA1c (7.3% vs 7.6%; p=0.0015).

The whole study population had been previously treated with MDI – Multiple Daily Injections (18.0%), SAP - Sensor Augmented Pumps (24%), PLGS – Predictive Low Glucose Suspend pumps (38%) or HCL – Hybrid Closed Loop pumps (20%).

There were no significant differences between the two groups at baseline when analyzing glycemic parameters; except for time of sensor use that was significantly higher in Control-IQ users (98% vs 92.9%; p=0.0005).

**Figure 1:**
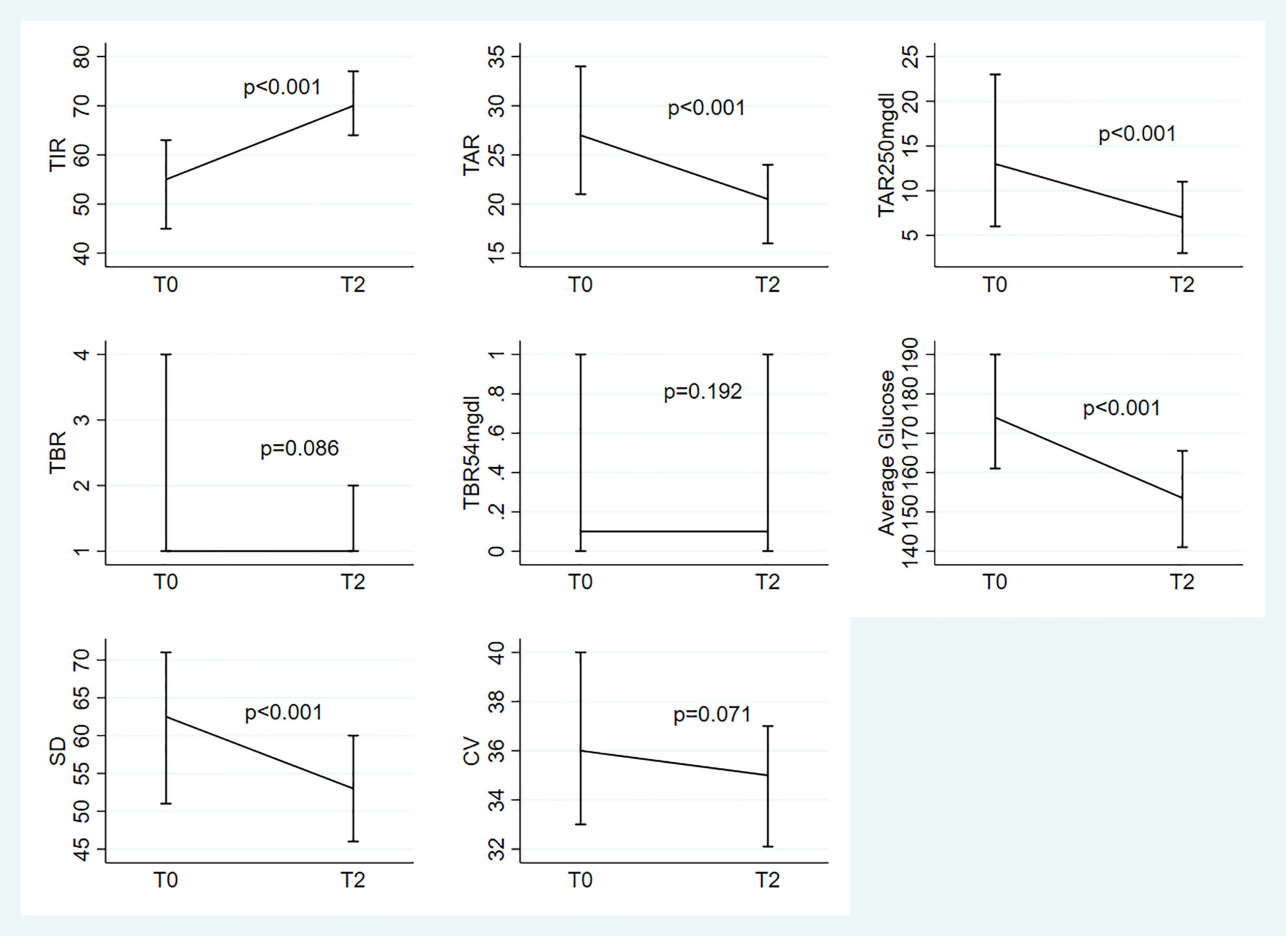
Median(IQR) at To and T2 for the 8 parameters under study; evaluation of the change from T0 based on the linear mixed models with random intercept adjusted for the following baseline variables: age, disease duration, HbA1c and type of previous treatment.

**Figure 2:**
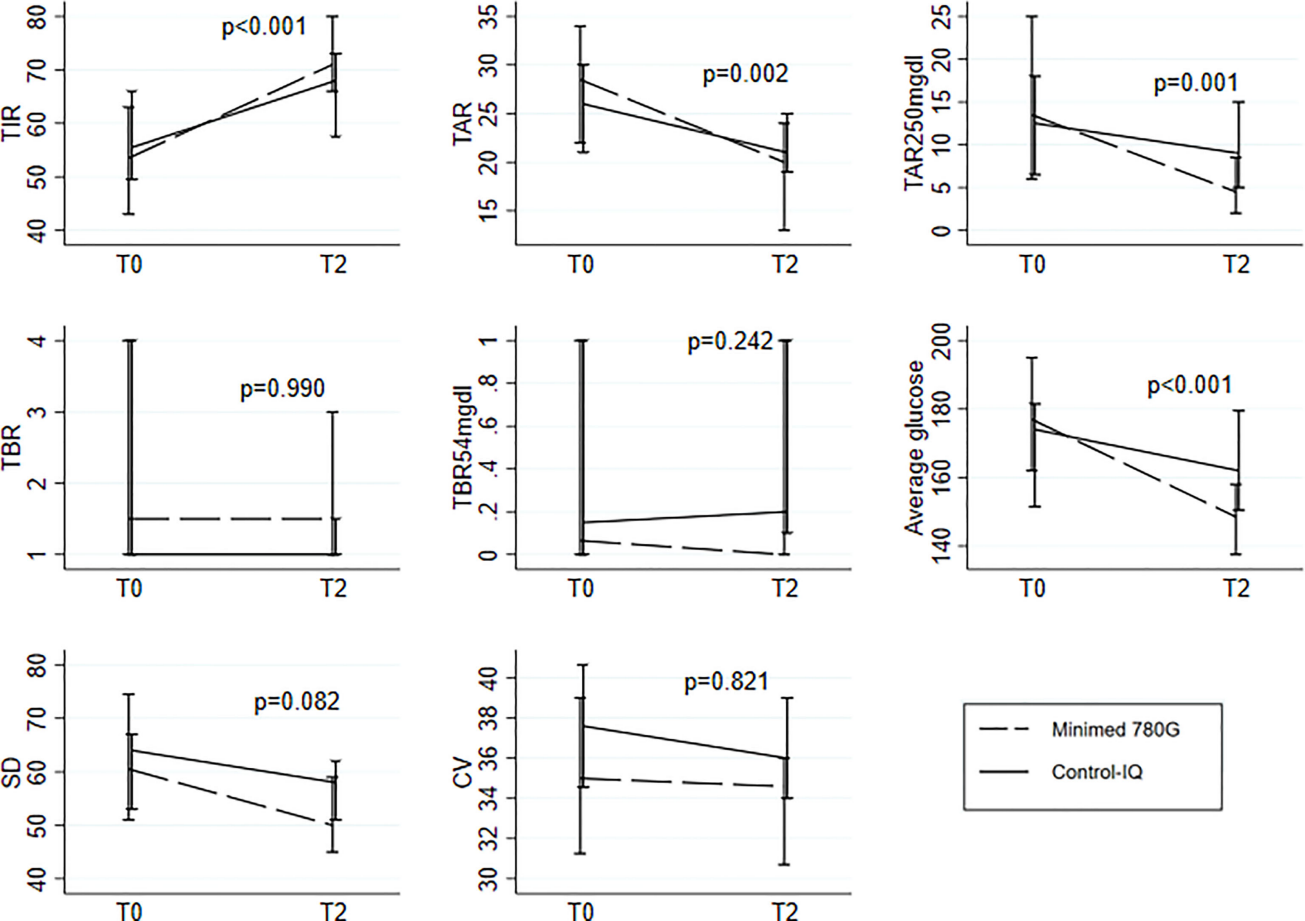
Median(IQR) at To and T2 for the 8 parameters under study separately for the two systems; comparison of the pattern change between the two groups testing the Time-Group interaction in the linear mixed models with random intercept. All the models were adjusted for the following baseline variables: age, disease duration, HbA1c and type of previous treatment.

The longitudinal comparison between the two devices is shown in **Figure 1** and in **Figure 2**.

We observed significant variations over time in TIR (p<0.001), TAR (p<0.001), TAR>250mg/dl (p<0.001), average glucose levels (p<0.001) and SD (p<0.001). “Almost significant” differences were observed for TBR and CV (respectively: p=0.086 and p=0.071). No significant variations were found for TBR<54mg/dL (p=0.192) (**Figure 1**).

The evaluation of ΔT0-T2 brought out the following significant differences between the two devices: MiniMed is more effective than Control-IQ in improving TIR (p<0.001), TAR (p=0.002), TAR>250mg/dl (p=0.001) and average glucose levels (p<0.001), No significant differences were found between the two devices for SD (p=0.082), CV (p=0.821), TBR (p=0.990) and TBR<54mg/dL (p=0.242) (**Figure 2**). As a sensitivity analysis, the interaction was also assessed within pediatric (age<18: N=38; Minimed 780G: N=18(47%), Tandem-Control IQ: N=20(53%)) and within adult (age>=18: N=36; Minimed 780G: N=24(67%), Tandem-Control IQ: N=12(33%)) patients. Results remained consistent between the two groups except for TAR (p=0.245 and p=0.006 respectively) (**Supplementary Table 1**).

The comparison of the two devices at T2 is illustrated in **Table 2**. Both devices improved glycemic control parameters. At T2 TIR is higher in MiniMed patients than in Control-IQ patients (71% vs 68%; p=0.001); TAR>250mg/dL (4.5% vs 9%; p=0.009) and TAR (20% vs 21%; p=0.001) are lower in MiniMed patients. Average blood glucose levels (148.5 mg/dL vs 162 mg/dL; p=0.001) and SD (50 mg/dL vs 58 mg/dL; p=0.031) are lower in MiniMed patients. There are no significant differences between the two groups when considering TBR, TBR<54mg/dL and CV.

**Table 2:** Treatment effects overall and by group at T2.

	Overall	Minimed 780G	Control-IQ	p-value
TIR (%), Median (IQR)	70 (64; 77)	71 (66; 80)	68 (57.5; 73)	0.001
TAR (%), Median (IQR)	20.5 (16; 24)	20 (13; 24)	21 (19; 25)	0.001
TAR>250mgdl (%), Median (IQR)	7 (3; 11)	4.5 (2; 8.5)	9 (5; 15)	0.009
TBR (%), Median (IQR)	1 (1; 2)	1.5 (1; 3)	1 (1; 1.5)	0.381
TBR<54mgdl (%), Median (IQR)	0.1 (0; 1)	0 (0; 1)	0.2 (0.1; 1)	0.447
Average glucose (mg/dl), Median (IQR)	153.5 (141;165.5)	148.5 (137.5;158)	162 (150.5;179.5)	0.001
SD (mg/dl), Median (IQR)	53 (46; 60)	50 (45; 59)	58 (51; 62)	0.031
CV (%), Median (IQR)	35 (32.1; 37)	34.6 (30.7; 36)	36 (34; 39)	0.620
Time Active CGM (%), Median (IQR)	96 (90; 98)	93.5 (85; 97)	98 (94; 99)	0.0001

TIR, Time in Range (70-180 mg/dl); TAR, Time Above Range (181-250 mg/dl); TAR250, Time Above Range (>250 mg/dl); TBR, Time Below Range (54-69 mg/dl); TBR54, Time Below Range (<54 mg/dl); SD, Standard Deviation; CV, Coefficient of Variation; CGM, Continuous Glucose Monitoring. Bold values indicates statistically significant.

## 4 Discussion

The aim of this study was to compare real-life glycemic control data between Minimed 780G and Tandem Control-IQ users one year after starting the system. To the best of our knowledge, this is the first study to compare efficacy and safety of the AHCL systems currently available in Italy in children and adults with T1D over such a long period of time.

Schiaffini et al. (31) carried out another study only on pediatric patients affected by T1D and compared the efficacy of Minimed 780G and Control-IQ in improving glycemic control 1-month after starting the devices. Their results are similar to ours when considering the rapidity with which AHCL devices improve glycemic control, but they don’t highlight significant differences between the two devices evaluated; therefore, according to their study, they appear to be equivalent after 1-month of therapy.

Considering our previous 1-month study, the results are only partially confirmed at one-year follow up (30): Minimed 780G still appears to be superior in managing hyperglycemia, whereas we couldn’t confirm Control-IQ’s superiority in reducing glycemic variability and hypoglycemic events. Minimed 780G is still more efficient in improving TIR and reducing average blood glucose levels. Both devices improve glycemic control significantly. Minimed 780G achieves all targets recommended by the International Consensus at T2, whereas Control-IQ is slightly below target when considering TAR>250mg/dl (9%) and TIR (68%). Minimed is significantly more efficient than Control-IQ when considering average blood glucose levels, TIR, TAR and TAR>250mg/dl.

Despite the differences between the two devices in terms of effectiveness, there is an important age difference and disease duration between the two groups. Control-IQ users are younger and have a shorter disease duration. This is inevitable considering that all Control-IQ users were followed by Giannina Gaslini Pediatric Institute Diabetology Center. Of course, this data must be considered while we discuss the results of the study, because childhood and adolescence are certainly more critical moments in the management of glycemic control than adulthood due to physiological (eg. hormonal changes) and environmental (eg. lifestyle) factors. Furthermore, a shorter disease duration could correspond to less-skilled patients in the management of T1D. The slight inferiority of Tandem in the improvement of glycemic parameters and in the targets obtained at T2 must also be considered in relation of this data.

Another interesting result to discuss regards the CGM use; MiniMed 780G is in disadvantage when compared to Control-IQ in terms of time of sensor use. Our interpretation of this result is based on the fact that MiniMed 780G users were all using Guardian 3 at the time of the study. Guardian 3 sensor requires capillary glycemia calibrations twice daily and SmartGuard (automatic mode) is deactivated by the system if no calibrations are performed. On the other hand, Control-IQ users use Dexcom G6 sensor that doesn’t require calibrations. This could mean that the Tandem users have had less possibility of deactivation of automatic mode (Control-IQ) than the Minimed users (SmartGuard). Minimed 780G could be even more effective in improving glycemic control using the new Guardian 4 sensor which does not require calibration to run the system in automatic SmartGuard mode.

Comparing the results of our previous 1-month study and this 1-year follow-up study, even if Minimed 780G appears to be more effective, especially over a longer period, both devices better improve glycemic control in the first month of treatment and then this tends to stabilize over the following months; glycemic parameters don’t improve ulteriorly, on the contrary they may even slightly worsen. This could be due to the fact that the patients were followed more attentively after positioning the new pump since follow-up visits are more frequent. Furthermore, positioning a new pump is a moment of great change for the patients, characterized by motivation, thrive to improve and major attention to treatment regimens and glycemic control. In our opinion, it is very important to reinforce the patient’s motivation to take better care of themselves and perform frequent retraining during the follow-up visits on the correct use of the devices and their functionality and potential. This constant reinforcement of patient education and technological support can be fundamental in maintaining the improvement in glycemic control achieved 1-month after starting the device and in creating possibilities for further improvement in glycemic control over time.

A limitation of our study is the number of the study population. Due to the nature of the extended study, no sample size calculation was performed since all the patients with available T2 data were included and the sample was smaller when compared to our first study. The 16 patients who weren’t included in this extended study continued using AHCL system but weren’t available for follow-up visits and data download at T2. Another limitation of this study is the heterogeneity between the two groups due to the nature of the study (no randomization). There are two main limitations concerning age: wide age-range of patients involved and the difference of age between the two groups. To take into account these design issues, we adjusted the models for baseline confounders and we presented a sensitivity analysis separately for pediatric and adult patients. However, further studies involving a greater number of patients and with a more uniform age and characteristics between the comparison groups are necessary. Furthermore, it would be interesting to evaluate the glycemic parameters obtained by Minimed 780G in association with the Guardian 4 sensor.

In conclusion, both AHCL systems improve glycemic control, even after just one month of treatment. After 1-month of AHCL use, further improvement in glycemic control was not observed. Minimed 780G is slightly superior to Tandem Control-IQ in improving glycemic control at 1-year follow-up.

## Data availability statement

The original contributions presented in the study are included in the article/**Supplementary Material**. Further inquiries can be directed to the corresponding author.

## Ethics statement

Ethical review and approval was not required for the study on human participants in accordance with the local legislation and institutional requirements. Written informed consent to participate in this study was provided by the participants’ legal guardian/next of kin.

## Author contributions

BM designed the study and wrote the manuscript, PL designed the study and wrote the manuscript, SI researched data, SF researched data and reviewed the manuscript, PM did statistical analysis, MN designed the study and contributed to the discussion, MD designed the study and contributed to the discussion. All authors contributed to the article and approved the submitted version.
