# Supplementary material for: One-year follow-up comparison of two hybrid closed-loop systems in Italian children and adults with type 1 diabetes
Source: Front Endocrinol (Lausanne). 2023 Jan 26;14:1099024. doi: 10.3389/fendo.2023.1099024 (PMC9909036; doi:10.3389/fendo.2023.1099024)
Supplement: Supplementary file 1 [file DataSheet_1.pdf]

Adults:						
	**Minimed 780G**			**Tandem-Control IQ**		
	**Pre**	**Post**	**Pre-Post change**	**Pre**	**Post**	**Pre-Post change**
**TIR**	52.92(15.65)	72.88(10.25)	19.96(15.45)	58.83(15.85)	62.83(14.89)	4.00(10.05)
**TAR**	32.58(9.83)	19.04(7.82)	-13.54(10.53)	27.08(9.70)	22.5(6.87)	-4.58(6.93)
**TBR**	2.72(3.13)	2.08(1.98)	-0.64(3.58)	1.35(1.60)	0.90(0.23)	-0.45(1.57)
Pediatrics						
	**Pre**	**Post**	**Pre-Post change**	**Pre**	**Post**	**Pre-Post change**
**TIR**	50.78(17.62)	73.11(9.41)	22.33(19.39)	57.85(13.48)	66.20(12.34)	8.35(17.57)
**TAR**	24.94(8.37)	18.44(6.55)	-6.50(8.65)	24.29(9.40)	20.85(7.04)	-3.44(8.02)
**TBR**	2.31(1.99)	2.06(1.76)	-0.25(2.18)	2.79(2.43)	2.15(2.55)	-0.64(1.59)

TIR – Time in Range (70-180 mg/dl)
TAR – Time Above Range (181-250 mg/dl)
TBR – Time Below Range (54-69 mg/dl)

Supplementary Table 1. Mean (SD) of TIR, TAR and TBR at T0, at T2 and for the T0-T2 change within adult and pediatric patients.
